# Decoding in the dark: extracting information from spontaneous activity in primary visual cortex

**DOI:** 10.1186/1471-2202-13-S1-P83

**Published:** 2012-07-16

**Authors:** Iñigo Romero Arandia, Ruben Moreno-Bote

**Affiliations:** 1 Foundation Sant Joan de Deu, Parc Sanitari Sant Joan de Deu, 08950 Esplugues de Llobregat, Barcelona, Spain

## 

Understanding the role of spontaneous activity in cortex is a crucial challenge, since it might be involved in sampling cortical states and in the preparation of the network for rapid responses to external stimuli. However, whether there is also information processing during spontaneous activity is still debated.

We asked how much information it is possible to extract from V1 spontaneous activity following visual stimulation. We analyzed multi-electrode (100) recordings in an experiment setup where a drifting grating with 8 possible motion directions was presented for 1s and was followed by a blank period of around 1s. Using maximum likelihood estimation (MLE) on a simple independent Poisson model, we found than spontaneous activity during a blank period allowed us to predict the stimulus orientation previously used with an accuracy of around 25% (100/8=12.5% chance level).

This result shows that spontaneous activity has in fact information about the preceding stimulus and establishes an information lower bound. Another crucial question is to know by how much we can improve the information that can be decoded. We observed that spontaneous activity is characterized by periods of high activity interleaving periods of low activity. Surprisingly, the average population vector during high activity periods was almost perfectly a scaled version of the population vector at low activity periods. This suggests a model where neurons are Poisson-like but their rates are modulated by a common mode. Applying this model to the data we could predict correctly 40% of the stimulus orientations.

Our results not only show that on average approximately half of the information provided during stimulation is still present in the subsequent blank period, but also suggest that global slow oscillations in spontaneous activity -well described by a multiplicative factor akin to the effect of attention to neuronal activity- are a critical component for information processing. Understanding the role of spontaneous activity in cortex is a crucial challenge, since it might be involved in sampling cortical states and in the preparation of the network for rapid responses to external stimuli. However, whether there is also information processing during spontaneous activity is still debated.

